# Significant Work Is About Self-Realization and Broader Purpose: Defining the Key Dimensions of Meaningful Work

**DOI:** 10.3389/fpsyg.2018.00363

**Published:** 2018-03-26

**Authors:** Frank Martela, Anne B. Pessi

**Affiliations:** Faculty of Theology, University of Helsinki, Helsinki, Finland

**Keywords:** authenticity, meaningful work, meaningfulness at work, purpose, calling, significance

## Abstract

Research on meaningful work has proliferated in recent years, with an increasing understanding of the centrality of meaningfulness for work-related motivation, commitment, and well-being. However, ambiguity around the main construct, “meaningful work,” has hindered this progress as various researchers have used partly overlapping, partly differing conceptualizations. To bring clarity to this issue, we examine a broad range of various definitions of meaningful work and come to argue that meaningfulness in the broadest sense is about *work significance* as an overall evaluation of work as regards whether it is intrinsically valuable and worth doing. Furthermore, we argue that there are two key sub-dimensions to this work significance: *Broader purpose* as work serving some greater good or prosocial goals (the intrinsic value of work beyond the person in question). And *self-realization* as a sense of autonomy, authenticity and self-expression at work (the intrinsic value of work for the person in question). Previous definitions of meaningful work feature typically one or two of these elements—significance, broader purpose, self-realization –, but in the future it would be beneficial to clearly acknowledge all three elements in both definitions and operationalizations of meaningful work.

## Introduction

Human beings are “hardwired to seek meaning” (Baumeister and Vohs, 2002, p. 613) and a lack of meaning is seen as a serious psychological deprivation associated with depression, mortality, and even suicide ideation (Harlow et al., 1986; Klinger, 1998; Steger et al., 2006; Tanno et al., 2009), especially in the context of late modernity and the pressure to live “authentically” (e.g., Giddens, 1991; Taylor, 1991). Given the importance of meaningfulness for human motivation and well-being (see e.g., Steger, 2012), and given the fact that in modern times work has become one of the key domains from which people derive meaningfulness (Baumeister, 1991; Steger and Dik, 2009), organizational researchers have increasingly turned their attention to studying what makes work meaningful. Having featured as a key psychological condition for job engagement (Kahn, 1990), a key cognitive element of empowerment (Thomas and Velthouse, 1990; Spreitzer, 1996), a central motivation for identity construction (Pratt, 2000; Pratt and Ashforth, 2003), and a core psychological state in the theory of job design (Hackman and Oldham, 1980), meaningful work has started to attract increased research attention as an important psychological state on its own (Pratt and Ashforth, 2003; Michaelson, 2005; Rosso et al., 2010).

With the development of validated scales to measure meaningful work (Lips-Wiersma and Wright, 2012; Steger et al., 2012a; Schnell et al., 2013), empirical research examining its antecedents and outcomes has also started to proliferate. As regards potential antecedents, conditions such as the socio-moral climate, work-role fit, (Schnell et al., 2013) and internal regulation (Allan et al., 2016) have been shown to be connected to meaningfulness of work. As regards potential outcomes, meaningfulness of work has been linked to occupational identification (Bunderson and Thompson, 2009), intrinsic work motivation, career commitment, (Steger et al., 2012a), affective well-being (Arnold et al., 2007), patient satisfaction in nursing (Leiter et al., 1998), and supervisor-rated performance (Harris et al., 2007). It is also associated with less work absenteeism (Steger et al., 2012a) and decreased turnover intention (Scroggins, 2008; Arnoux-Nicolas et al., 2016).

Furthermore, besides these associations, the tendency to find meaning in work has been illustrated to have various long-term effects too; e.g., experiencing work as meaningful prospectively predicts whether employees felt that they derived some psychological benefits from a stressful work-related event (Britt et al., 2001). Based on this emerging awareness of the importance of meaningful work for work-related motivation, commitment and overall well-being (Rosso et al., 2010; Lepisto and Pratt, 2017), meaningful work has the potential to become a key research topic in future studies of the psychological underpinnings of the work experience.

However, as many have noted (see Steers et al., 2005; Rosso et al., 2010; Lepisto and Pratt, 2017), research on the topic suffers from definitional ambiguity as regards what is meant by the main construct, “meaningful work”^1^. For some, it is simply a judgment of the work being “significant” (Bunderson and Thompson, 2009, p. 40), while others see it as being about pursuing a purpose more important than money (Sparks and Schenk, 2001, p. 858), and still others see it as being about a “sense of return of investments” of one's self in terms of physical, cognitive or emotional energy (Kahn, 1990, p. 705). It has also been connected to, for instance, values and having a “morally worthy work” (Ciulla, 2000, p. 225), and even to work where the humanity in an employee is treated as an end and not as a mere means (Bowie, 1998). A critical look at the various definitions of meaningful work given in the literature makes it clear that the concept “will need to be clarified” (Steers et al., 2005, p. 238) as there are “fundamental differences in how meaningfulness is conceptualized” (Lepisto and Pratt, 2017, p 101). Furthermore, the lack of consensus as regards the nature of meaningful work leads to the danger of conflating meaningful work with its antecedents and outcomes (cf. Constantini et al., 2017) and is reflected also in the fact that more often than not empirical studies tend to come up with their own measures for meaningful work (e.g., Britt et al., 2001; Sparks and Schenk, 2001; Arnold et al., 2007; Bunderson and Thompson, 2009) instead of using established measures (to which we return later), making it hard to compare various findings. Given the differences in how meaningful work is understood, conceptualized, and operationalized, there is a real danger that the theoretical and empirical works will talk past each other leading to confusions and misunderstandings. Before these fundamental differences in conceptualizations of meaningful work are reconciled, it is difficult to make any theoretical or empirical progress in investigations of meaningful work. This challenge is the core motivation for the present article.

More specifically, we will review a broad number of definitions of meaningful work by various researchers in order to identify key themes and recurring elements. Based on this review, the main target of the present article is to, firstly, suggest that there are three separate dimensions to which the various researchers typically refer to when talking about meaningful work: significance, broader purpose, and self-realization. Secondly, in addition to identifying these three separate dimensions, we aim to advance theory by offering a suggestion on how they are related to each other. Essentially, we argue that significance is the broadest way of understanding what meaningful work means; it is about whether the work has some intrinsic value. Self-realization and broader purpose, in turn, are the two types of intrinsic value or the two ways through which work can be significant. We compare this way of understanding meaningful work with other prominent suggestions in the literature, aiming to show why we don't see some previously suggested pathways to meaningful work as foundational. Although several reviews of meaningful work have been published (e.g., Chalofsky, 2003; Cheney et al., 2008; Rosso et al., 2010), a review that would concentrate on the *definitions* of meaningful work has not yet been conducted (Lepisto and Pratt, 2017 come closest, and we'll return to the key differences between their and our conclusions). All in all, the definitional separation of meaningful work into three elements that the article at hand aims to accomplish is crucial in clarifying the construct, and making it possible to distinguish the proposed dimensions of meaningful work both theoretically and—in the future—empirically.

### Meaning as descriptive, meaningfulness as evaluative

Building up toward an understanding of meaningful work, we should start by understanding what “meaning” as such means. That is, what is the meaning of meaning? On the most fundamental level, meaning is about forming mental representations of the world that aim to identify possible relationships among various phenomena (Baumeister, 1991; Heine et al., 2006; Martela and Steger, 2016). Finding meaning is about connecting; meanings are the expected relationships and associations that human beings see in their world. In this sense meanings are constructed, they are something we impose upon the world (Baumeister and Vohs, 2002). In other words, our ways of looking and understanding the world are much determined by the *meaning frameworks* we have acquired socially, societally, and culturally. These meaning frameworks are “complex web of propositions that we hold about how things are in the world and how things will be” (George and Park, 2016, p. 206). These meaning frameworks—also referred to as *meaning systems* (e.g., Silberman, 2005)—are the cognitive tools we use to navigate and operate in the everyday; they help us to make sense of our current experience, give us direction about what goals and aims to pursue, and guide us about what is valuable and what really matters in life and in the world (George and Park, 2016). Thus, “people structure and interact with the world differently on the basis of the meaning they assign to events in their social and physical environments” (Molden and Dweck, 2006, p. 192). We acquire these meaning frameworks mainly from two sources: They are partly built up from the generalizations we make from our own past experiences, but at the same time they are highly influenced by our society, culture and upbringing from which we acquire much of our vocabularies, values and ways of making sense of the world.

Different people attach different meanings to their work. Some might see it as a mere means of getting a paycheck, while others see it as a game of status and promotions leading to a successful career. Still others view their work as a calling, the work being its own fulfillment (Bellah et al., 1985; Wrzesniewski et al., 1997). However, the question about the *meaning* of work is different from the question of the *meaningfulness* of work. While meaning is “the output of having made sense of something”, meaningfulness is about “the *amount* of significance” one attaches to something (Rosso et al., 2010, p. 94). While meaning is a *description* of how one understands what work means, meaningfulness is thus a specific type of *evaluation* or experience (Martela and Steger, 2016, p. 536). Accordingly, when Tummers and Knies (2013) define work meaningfulness as “an employee's perception that he or she is able to understand the complex system of goals in the organization and its relationship to his or her own work,” they seem to refer more to meaning than to meaningfulness of work. The experience of having a sense of meaningfulness is both a cognitive and an emotional assessment about the presence of purpose and value in one's life or in one's work constructed both socially and individually (Wong, 1998; Park, 2010). Meaningfulness—for humans—is about what guides, directs and gives value to our endeavors (Frankl, 2006/1946).

Indeed, when we say that our work is meaningful, we are not referring to our way of conceptualizing work but rather are making an evaluation of it, or stating that we derive a certain kind of experience from it. The meaningfulness we derive from work might be based on a certain meaning we attach to work; for instance, a person seeing one's work as a calling might get more meaningfulness out of it than a person seeing it as a mere job (see e.g., Bunderson and Thompson, 2009). Accordingly, despite a similar concept, *meaningfulness* of work and *meaning* of work are two separate issues. As Ciulla (2000, p. 224) puts it, “we not only make sense of the world, we assign significance to it.” Meaning is *descriptive*, it tells us about the specific meaning framework one attaches to work, while meaningfulness is *evaluative*, it is an evaluation of one's work based on how well it fulfills certain values or characteristics.

Furthermore, when we talk about meaningfulness of work, we talk about a subjective experience or evaluation. As will be evident in our review of various definitions of meaningful work (see Table 1), there is a wide agreement that meaningful work is a subjective experience rather than some kind of objective characteristic of work itself. Rosso et al. (2010) made the same observation in their review of work, noting that organizational researchers have “primarily employed a psychological perspective” in discussing meaning of work. Even the business ethicists discussing the objective conditions for meaningful work tend to see that the employer's moral responsibility to provide certain objective conditions is based on the fact that providing these conditions makes it possible for the individual to *experience* subjective meaningfulness at work (Michaelson, 2011). Accordingly, meaningful work is taken to be something subjective; an experience, a feeling or an evaluation of one's work. Meaningfulness as an experience thus seems to involve both affective and cognitive elements. Our view is that meaningfulness is primarily a type of feeling we have when we work or a feeling that arises when we think about our work. Work *feels* or *doesn't feel* meaningful. When we are asked to cognitively evaluate whether our work is meaningful, we thus would typically look for how strong this feeling is present in our recollected experiences of work. Such a subjective interpretation of meaningfulness connects meaningful work to psychological research on meaning in life: both are about the experience of meaningfulness, the former being about the meaningfulness one experiences as regards to one's work, the latter being about the meaningfulness one experiences as regards one's whole life.

**Table 1:** The definitions of meaningful work in various sources and our interpretation of which of the three dimensions are referred to in the given definition. “Other' means that the definition refers to a dimension other than the three we concentrate on.

	**Construct**	**Definition**	**Context**	**Purpose**	**Significance**	**Self-realization**	**Other**
Hackman and Oldham, 1975, p. 162	Experienced meaningfulness of the work	“The degree to which the employee experiences the job as one which is generally meaningful, valuable, and worthwhile”	Job design theory		X		
Schwartz, 1982, p. 640	Meaningfully structured work	“Arranged to allow all persons to act as autonomous agents while performing their jobs”	Philosophical ethics			X	
Arneson, 1987, p. 517	Meaningful work	“Work for which pay is received is interesting, calling for intelligence and initiative, and in which the worker has considerable freedom to determine how the work is to be done and a genuinely democratic say over the character of the work process and the policies pursued by the employing enterprise”	Philosophical ethics			X	X
Kahn, 1990: pp. 703–704	Psychological meaningfulness	“A feeling that one is receiving a return on investments of one's self in a currency of physical, cognitive, or emotional energy”	Employee engagement			X	
Walsh, 1994, pp. 243–244	Meaningful work	“Work which offers opportunities for eudaimonian activity” that involves “the development of skills and capacities”	Philosophical ethics				X
Renn and Vandenberg, 1995, p. 282	Experienced meaningfulness of the work	“The extent to which an individual believes his or her job is important vis à vis the individual's own value system”			X		
Bowie, 1998, p. 1087	Meaningful work	“Meaningful work is work that is freely entered into, that allows the worker to exercise her autonomy and independence, that enables the worker to develop her rational capacities, that provides a wage sufficient for physical welfare, that supports the moral development of employees and that is not paternalistic in the sense of interfering with the worker's conception of how she wishes to obtain happiness”	Kantian ethics			X	X
Ciulla, 2000: 225	Meaningful work	“Morally worthy work undertaken in a morally worthy organization”	Nature of modern work	X			
Britt et al., 2001, p. 55	Meaningful work	“(a) Being engaged in important and relevant work [--] and (b) experiencing events [at work] that put [the work] in a broader contextual framework”	Stressful events and work trauma	X	X		
Sparks and Schenk, 2001, p. 858	Belief in a higher work purpose	“Purposes ‘more important’ than simply making money”	Transformational leadership	X			
Sarros et al., 2002, p. 287	Meaninglessness	“Inability to comprehend the relationship of one's contributions to a larger purpose“	Work alienation	X			
Chalofsky, 2003, p. 74	Meaningful work	”That which gives essence to what we do and what brings a sense of fulfillment to our lives“	Meaningful work as such		X		X
Pratt and Ashforth, 2003, p. 311	Meaningful	”Work and/or its context are perceived by its practitioners to be, at minimum, purposeful and significant”	Meaningful work as such	X	X		
May et al., 2004, p. 14	Experienced meaningfulness	“The value of a work goal or purpose, judged in relation to an individual's own ideals or standards”	Employee engagement	X	X		
Arnold et al., 2007, p. 195	Meaningful work	“Finding a purpose in work that is greater than the extrinsic outcomes of the work”	Transformational leadership	X			
Cheney et al., 2008, p. 144	Meaningful work	“Work that contributes to a personally significant purpose”	Organizational communication	X	X		
Grant, 2008, p. 119	Experience of meaningfulness	”A judgment of the general value and purpose of the job“	Task significance	X	X		
Berg et al., 2009, p. 974	Personal and social meaning	[perceiving work] ”as intrinsically enjoyable and as making valuable contributions to society”	Callings	X		X	
Bunderson and Thompson, 2009, p. 40	Sense of work meaning	“My work is significant”	Neoclassical callings		X		
Lieff, 2009, p. 1384	Meaningful work	“The realization of one's potential and purpose purpose—the point at which a person's passions, strengths, and core values interact synergistically in his or her work”	Career development in medicine	X		X	
Rosso et al., 2010, p. 95	Meaningful work	“Work experienced as particularly significant and holding more positive meaning for individuals”	Meaningful work as such		X		
Fairlie, 2011, p. 510	Meaningful work	“Job and other workplace characteristics that facilitate the attainment or maintenance of one or more dimensions of meaning”	Human resource development				
Beadle and Knight, 2012, p. 433	Meaningful work	Work “ordered toward goods of excellence pursued within social practices”	Virtue ethics		X		
Steger et al., 2012a, p. 323	Meaningful work	“Work that is both significant and positive in valence”	Meaningful work as such		X		X
Lips-Wiersma and Wright, 2012, p. 657	Meaningful work	“An individual subjective experience of the existential significance or purpose of work”	Meaningful work as such	X	X		
Roessler, 2012, p. 88	Meaningful work	”Being able to realize his talents and abilities, his “individuality,” in the work and the producing activity in a self-determined way“	Philosophical ethics			X	
Hirschi, 2012, p. 480	Work meaningfulness	”The amount of significance people perceive in their work“	Callings		X		
Tummers and Knies, 2013, p. 861	Work meaningfulness	”An employee's perception that he or she is able to understand the complex system of goals in the organization and its relationship to his or her own work“	Leadership in public sector				X
Berg et al., 2013	Meaningfulness	“The amount or degree of significance employees believe their work possesses”	Job crafting		X		
Schnell et al., 2013, p. 543	Meaning in work	”A sense of coherence, direction, significance, and belonging”	Meaningful work as such	X	X		X
Chalofsky and Cavallaro, 2013, p. 332	Meaning one finds in work	“The extent to how much the work reflects who we are”	Meaningful work as such			X	
Allan et al., 2014, p. 545	Meaningful work	“The subjective experience that one's work has significance, facilitates personal growth, and contributes to the greater good”	Meaningful work as such	X	X	X	
Raub and Blunschi, 2014, p. 11	Meaningful work	“Requires that employees understand the significance of what they do”	Corporate Social Responsibility		X		
Yeoman, 2014, p. 249	Meaningful work	Work “constituted by the goods of autonomy, freedom, and dignity”	Business ethics		X	X	
Lepisto and Pratt, 2017	Meaningful work	“(a) Realizing one's self through work, and (b) being able to account for worth of one's work”	Meaningful work as such		X	X	
Bailey et al., 2017	Meaningful work	“Work that is personally enriching and that makes a positive contribution”	Meaningful work as such	X		X	

### The three meanings of meaningful work: significance, self-realization, broader purpose

Our analysis of the definitions of meaningful work proceeded through three steps: First, we reviewed the literature aiming to identify how various authors have defined meaningful work. We didn't aim to make a comprehensive review, but rather wanted to identify the most influential and cited definitions. Accordingly, in addition to work that we already were aware of, we searched in the ISI Web of Science Core Collection and in the Scopus for all articles with “meaningful” or “meaningfulness” and “work” in the title that had received over 10 citations in either of the collections. In addition to looking at the definitions given in these articles, we also looked for any work that these articles cited when giving their definition as well as for other work that was identified as especially interesting as regards this topic. We also examined recent reviews (Rosso et al., 2010; Lepisto and Pratt, 2017) to identify other articles of interest. Altogether we reviewed 61 articles, and while some articles didn't include any clear definition of meaningful work (e.g., Strong, 1998; Horowitz et al., 2003; Michaelson, 2005; Dik et al., 2009; Leufstadius et al., 2009; Sayer, 2009; Dempsey and Sanders, 2010; Jelinek and Ahearne, 2010; Lee and Carter, 2012; Michaelson et al., 2014) and others referred directly to an existing definition covered in this review (e.g., Lips-Wiersma and Morris, 2009; Pavlish and Hunt, 2012; Schnell and Hoof, 2012; Steger et al., 2012b; Munn, 2013; Rothmann and Hamukang'andu, 2013; Geldenhuys et al., 2014), we were able to identify 36 separate definitions of meaningful work, which are listed in Table 1.

Based on an examination of this literature, we identified the most typical, most frequently used elements in the definitions^2^. It quickly became clear that there were three such elements: significance, broader purpose, and self-realization. Then we systematically examined each definition found in the reviewed articles and coded whether it referred to one or more of these three elements. We also marked down if it included some other elements. This information is displayed in Table 1.

Based on our review and utilizing also broader literature on meaning in life, we aim to offer both a deeper understanding of what is meant by each of the three dimensions, and a proposal about how they are interlinked. Thus, the analysis was not purely inductive, data-determined nor deductive, theory-based, but could be characterized as abductive (Martela, 2015). We will next look at each of the three dimensions in turn, aiming to offer a proper examination of their nature and how they should be defined, and how they have been featured in previous definitions of meaningful work.

### Significance

Starting with significance, we define it—based on both our review at hand and wider literature—as being about how much intrinsic value people assign to or are able to find from their work. In many definitions of meaningful work, the construct comes down to this overall sense of intrinsic value and worthwhileness of work. For example, in their theory of job design, Hackman and Oldham (1980) establish meaningfulness of work as one of the core psychological states, defining it as “the degree to which the employee experiences the job as one which is generally meaningful, valuable, and worthwhile” (Hackman and Oldham, 1975, p. 162). Similarly, for Berg et al. (2013) meaningfulness is about “the amount or degree of significance employees believe their work possesses,” for Raub and Blunschi (2014, p. 11) it is about employees understanding “the significance of what they do,” while Rosso et al. (2010, p. 95) define meaningful work as “work experienced as particularly significant and holding more positive meaning for individuals,” thus also putting the exclusive emphasis on significance. For Bunderson and Thompson (2009, p. 40) work meaningfulness seems to be simply about the experience that “my work is significant.” This intrinsic value of work is also reflected in Renn and Vandenberg (1995, p. 282) definition of meaningful work as “the extent to which an individual believes his or her job is important vis à vis the individual's own value system.” This comes close to Beadle and Knight's (2012) Aristotelian account of meaningful work, where it is about the possibility to pursue and realize the goods and values internal to the specific practice. This dimension is also directly connected to research on meaning in life, where significance has similarly been identified as one of the main ways meaningfulness is understood, defined as a “sense of life's inherent value and having a life worth living” (Martela and Steger, 2016, p. 534). Similarly, applied to work, significance is about a sense of work's inherent value and having a work worth doing.

Furthermore, in addition to these writers for whom meaningfulness is exclusively about significance, there are several definitions of meaningful work—especially in the last 10 years (see Table 1)—where “significance” or “general value” of work is one of the key components of meaningful work (e.g., Pratt and Ashforth, 2003; Grant, 2008; Lips-Wiersma and Wright, 2012).

This significance perspective on meaningful work is eloquently elaborated by Lepisto and Pratt (2017) in their recent review of meaningful work. Calling it the *justification perspective* they see it as based on people's need to “develop an account or justification regarding why their work is worthy or valuable” (Lepisto and Pratt, 2017, p. 106). The value of one's work can come to be questioned in two ways: Either the individual holds certain values but feels that one's work is not connected at all to these values, or—and this is a modern malady sociologists have been talking about (Weber, 1958; Bellah et al., 1985)—the individual feels uncertainty and separation from any values that could be used to justify the worthiness of one's work. Lepisto and Pratt refer to the latter with the classical sociological concept of *anomie*, a feeling of pointlessness seeing it as a core problem for which accounts of significance are a remedy. Account-making is for them the activity “where individuals seek to justify their work as possessing positive worth” (Lepisto and Pratt, 2017, p. 109). In such account-making we are thus seeking a point for our existence that goes beyond mere survival. Instead of merely staying alive, we are aiming to answer the classical existential question that tormented Tolstoy too in his later years to the point of constantly contemplating suicide: “Why should I live?” (Tolstoy, 2000, p. 17.) Significance of work is thus about aiming to find some intrinsic value in one's work-related activities that make them worth doing, it is a general evaluation of “the value or worth of one's work” (Lepisto and Pratt, 2017, p. 106).

### Broader purpose

Broader purpose, in turn, is connected to the idea that the work must contribute to some “greater good”, something beyond individual's own benefits. The core idea is that work should somehow contribute to self-transcendence: being part of or serving something bigger, greater that the individual oneself values. That is, the purpose in question of work must be something “greater than the extrinsic outcomes of the work” (Arnold et al., 2007, p. 195) or “more important” than simply making money“ (Sparks and Schenk, 2001, p. 858). Purpose as used in connection to meaningful work seems not to refer to mere purpose as a sense of directedness in life, but rather to “higher” or “greater” purpose where the directedness is directed at something larger than one's own benefits. For example, Sarros et al. (2002, p. 287) see that meaninglessness is about the “inability to comprehend the relationship of one's contributions to a larger purpose.” Berg et al. (2009, p. 974), in turn, see personal and social meaning as being partly about “making valuable contributions to society” and similarly for Steger et al. (2012a, p. 326; see also Allan et al., 2014) one principal facet of meaningful work is “the desire to positively contribute to the greater good.” In her historical treatment of how we understand work, Ciulla (2000, p. 225) comes to define meaningful work as “morally worthy work undertaken in a morally worthy organization.” Having a morally worthy work means for her that “there is some good in it” with the most meaningful works being those where people “directly help others or create products that make life better for people” (p. 225).

Thus, while the *purpose of working* might for many people be about getting a salary, we don't find people saying that salary is what makes their work *purposeful*. Broader purpose or purposefulness can thus be defined as a sense that through one's work one is serving something valuable beyond oneself, usually other people. Thus Rosso et al. (2010, p. 111) see that purpose is other-oriented rather than self-oriented and results “from participating in a larger system of shared values” rather than being about “one's personal values or interests.” And given the goal-oriented nature of purpose as a construct (McKnight and Kashdan, 2009), it is about advancing these broader values through one's actions. In other words, having a purposeful work means that one believes that one is able to have a positive impact in the wider world through one's work (Martela and Ryan, 2016a). This positive impact can be about grand goals such as fighting diseases, bringing forth political change or saving the environment, but it can also be more everyday positive impact such as helping one's customers or making one's clients happy.

Furthermore, it is important to understand that the broader purpose one serves through one's work can also be realized by serving one's family. Especially in situations where income is scarce, a person might be strongly motivated to provide for the family, and this broader purpose might make even an otherwise tedious work motivating and meaningful. This is well demonstrated by a recent study by Menges et al. (2017), where they looked at the motivation and performance of low-income employees of a Mexican manufacturing company, showing that family motivation can strengthen the energy and performance of employees. The broader purpose one serves through one's work can thus take many forms from helping the customers or improving the society to supporting one's family.

### Self-realization

Finally, based on our review we concluded that *self-realization* should be seen as the third separate dimension of meaningful work. It is about self-connectedness, authenticity, and how much we are able to realize and express ourselves through our work. Chalofsky and Cavallaro (2013, p. 332), for example, see that meaning as applied to work “has to do with the extent to how much work reflects who we are,” and Kira and Balkin (2014) make a close association between personal meaningfulness and the alignment between one's work and identity. For Lieff (2009, p. 1384) meaningful work is similarly about “the realization of one's potential and purpose,” in other words “the point at which a person's passions, strengths, and core values interact synergistically in his or her work.” Also Fairlie (2011, p. 510)—whose definition of meaningful is slightly circular in being about work that facilitates the “attainment or maintenance of one or more dimensions of meaning”—emphasizes that “self-actualizing” and “realizing one's full potential” are themes explicit in meaningful work. Rosso et al. (2010, p. 108), in turn, define authenticity as “a sense of coherence or alignment between one's behavior and perceptions of the “true” self.” They see that feelings of meaningfulness result from the fulfillment of this “central underlying self-motive.” Personal growth, which for example Steger et al. (2012a; see also Allan et al., 2014) mention as something that meaningful work may facilitate, could also be seen as one aspect of the more broader construct of self-realization.

The sense of autonomy and self-realization as the basis of meaningful work has been especially emphasized by researchers looking at meaningful work from an ethical perspective. For example, Schwartz (1982, p. 640) argues that meaningfully structured work is about allowing “all persons to act as autonomous agents while performing their jobs,” while Yeoman (2014, p. 249) argues that meaningful work is constituted by “autonomy, freedom, and dignity.” Roessler (2012, p. 88), in turn, argues that lack of self-realization leads to alienation, and accordingly meaningful work is about one “being able to realize his talents and abilities, his “individuality,” in the work and the producing activity in a self-determined way.” For these business ethicists and philosophers, offering meaningful work is a moral duty of the employer (Michaelson, 2005; Michaelson et al., 2014). Thus in their definitions of meaningful work they include a number of conditions that aim to ensure the autonomy of the employee by providing him with “considerable freedom to determine how the work is to be done” (Arneson, 1987, p. 517) and “that allows the worker to exercise her autonomy and independence” (Bowie, 1998, p. 1087). More broadly, authenticity has been defined as “realizing personal potential and acting on that potential” (Starr, 2008, p. 55, see also Pessi, 2013).

Lepisto and Pratt (2017) discuss this dimension as the *realization perspective* on meaningful work. For them it is about the “fulfillment of needs, motivations, and desires associated with self-actualization” (p. 104). They contrast it with a sense of alienation that arises when narrow and constraining work conditions leads to a sense of disconnection between oneself and one's actions. When one feels that one is just a “cog in a machine” doing something repetitious with no possibility to influence the content of one's work and constantly controlled by some authority, one might find the work not worth doing, even if it would be well compensated and have a noble purpose. In order for the work to be worth doing—instead of the worker feeling alienated,—the work thus has to be somehow connected to one's sense of who one is and give room for autonomy.

### How are the three dimensions of meaningful work connected?

All in all, we have three separate constructs: significance, broader purpose, and self-realization, and we can find authors claiming that each of these dimensions is what meaningful work is all about (see Table 1). However, instead of arguing that one of them is the “true” definition of meaningful work, we argue here that all three should be recognized as playing an essential role in people's definitions of meaningful work. In other words, if we really want to understand what phenomenon various authors have aimed to capture through the construct of meaningful work, we must recognize the presence of all three of these dimensions instead of relying on only one or two of them. They all seem to present a valid and unique angle to what meaningful work is about, and thus should not be ignored or brushed aside. While some authors, most notably Lepisto and Pratt (2017), have identified the importance of two of these dimensions (significance and self-realization), no previous work to our knowledge has acknowledged all three simultaneously. Thus, given our argument in the previous section that they all should be recognized as fundamental dimensions of meaningful work, recognizing this trichotomy in the definitions of meaningful work can serve to integrate previously separate pieces of scholarship.

However, beyond recognizing the separateness yet importance of all three dimensions of meaningful work, we aim to offer a proposal about how they relate to each other. More specifically, building on recent work that separates various intrinsic values from each other (e.g., Haybron, 2008; Martela, 2017a,b) we will argue that *significance* should be identified as the hallmark of meaningful work, operating on a more general level as compared to self-realization and broader purpose. This is because significance—as the general sense that work has intrinsic value and is worth doing—is the broadest possible evaluative question that can be asked about one's work, and similarly about one's life (Martela and Steger, 2016). Instead of looking at some specific characteristics of work—whether it is valuable *from the point of view* of offering possibilities for self-actualization for the worker or *from the point of view* of contributing to some greater good, one asks whether the work is valuable and worth doing *taken all into account*; whether work is valuable *an sich*.

Still, a further clarification needs to be made: Work has certain instrumental benefits, notably the salary one gets from it that helps to pay the bills^3^. But while one common *meaning* work can have for people is about making money, the starting point for many discussions of *meaningful* work is that in order to be meaningful, something deeper, “more important” than money must be present (Sparks and Schenk, 2001, p. 858). For example, a qualitative study of zookeepers noted that those with a sense of calling were “more willing to sacrifice money, time, and physical comfort or well-being for their work” (Bunderson and Thompson, 2009, p. 52). Similarly, philosophical discussions on meaningful life in general start with the assumption that a life worth living is more than mere survival. Thus Camus (1955/2000, p. 94) in a famous passage proclaims that “judging whether life is or is not worth living amounts to answering the fundamental question of philosophy.” The same concern is found in the core existential questions of world religions too (see e.g., Pessi, 2017). Work as a mere means for survival and sustenance is thus not enough to make it meaningful. Meaningfulness of work is about those motives and values that go beyond mere survival.

That is, being able to get bread to the table—and indeed having a table—might be an important reason to work, but in asking about the meaningfulness of work we are asking about the reasons to work beyond the mere extrinsic benefits that work provides. This can be illustrated by looking at Blake Fall-Conroy's artwork *Minimum Wage Machine*, which is basically a machine with a metal crank (Tech Times, 2015). When a participant turns the crank, a penny comes out every 4.5 s, leading the participant to earn $8 per h, the minimum wage of New York State at the time of the introduction of the art piece. Even though one gains the same amount of money as many jobs offer, turning the crank—that has no other purpose than dispensing the pennies—is arguably a prototype of meaningless work. A person devoid of any income might have a strong incentive to turn the crank—hunger, for example—but still (s)he would consider the work itself totally meaningless, a mere mean for getting the necessary pennies to pay for a decent meal. Significance is thus about whether work is valuable and worth doing for reasons other than its extrinsic benefits. Is the work valuable and worth doing based on its intrinsic qualities?

Given this broad and generic nature of *significance* as an overall evaluation of the value of work, we want to argue that we should see a *broader purpose* and *self-realization* as a way to break significance into two dimensions: One being more about the *intrinsic value* of work *beyond* the person in question, and the other being about the *intrinsic value* of work *for* the person in question. In other words, work can have intrinsic value for both the person oneself, and beyond the person, and this is captured by the concepts of self-realization and broader purpose. For example, Bailey et al. (2017) define meaningful work as “work that is personally enriching and that makes a positive contribution” implicating the importance of both personal realization and enrichment and serving a broader purpose. We want to argue that broader purpose and self-realization are two key types of significance.

Starting with the relation between broader purpose and significance, we suggest that in making the judgment of whether my work is worth doing, one of the major things we look at is whether the work serves some greater good or purpose. If we find such a purpose, this alone can make our work significant and worth doing (see Menges et al., 2017). Accordingly, research has found that helping others increases one's sense of meaningful work (Allan et al., 2017). Furthermore, research on meaning in life has found that prosocial behavior and a sense of prosocial impact (i.e., broader purpose) are closely connected to our evaluations of general meaningfulness (Martela and Ryan, 2016b; Tongeren et al., 2016; Pessi, 2017). Most importantly, having a prosocial impact has been connected to evaluations of significance (Martela et al., 2017). Significance is thus the broader category, being about all the ways that work can be intrinsically valuable, and purpose is taken as one of the two sub-dimensions within significance.

The argument for keeping significance and purposefulness separate is strengthened by the fact that purposefulness is not the only element that can make work valuable. We can namely argue that the significance of work is not only about *others* and how much we are able to contribute to them. It is also about *ourselves*, and how much we are able to realize ourselves. As humans, we need to feel that our existence matters—that our unique selves matter in this world (George and Park, 2014). We have the need to experience that our work aligns with our self-image, our view of who we are, our experience of authenticity, our values and interests. In other words, besides broader purpose, self-realization is another separate intrinsic value for us. This is true at least in the late-modern individualism-oriented societies (Taylor, 1991), but arguably sense of authenticity and autonomy are intrinsic values and key sources of well-being also in other, more traditional, collectivistic societies (Chirkov et al., 2003).

Self-realization, being true to oneself is actually often taken as one of the highest goals of an individual in late modernism (Taylor, 1991), and accordingly, we evaluate various life contexts, including work, by the extent to which it offers possibilities for such authenticity and self-realization. As Baumeister (1991, p. 124) notes, for many, work has become “the quintessential place to express and cultivate the self.” Accordingly, we argue that, along with purpose, self-realization is the other key dimension that makes work feel significant and worth doing. Both are intrinsic values that go beyond the mere extrinsic benefits of work, and together they cover both self-related intrinsic value of work as well as other-oriented intrinsic value of work.

Thus, we argue for an understanding of meaningful work, where significance is the overall judgment of the worthiness of work, and self-realization and broader purpose are the two key dimensions or two separate types of intrinsic values we look at in making such an overall judgment. Next, we will discuss how this proposition compares to a few key previous conceptualizations of meaningful work.

### Are there other dimensions to meaningful work?

Having defined meaningful as involving two sub-dimensions that together make up an overall evaluation, it is important to ask whether there could be other independent dimensions of work significance beyond broader purpose and self-realization. Here the discussion of the four major pathways to meaningful work by Rosso et al. (2010) offers an useful comparison. One of their pathways is *self-connection*, which they see as being about authenticity, self-concordance and being in close alignment with how one sees oneself. This is thus closely aligned with what we have here called self-realization. Similarly, *contribution* as a pathway is for them about perceived impact of one's work and doing work in the “service of something greater than the self” (p. 115). This thus comes close to what is here called the broader purpose. The difference is that while they see these two as pathways to meaningful work—and thus outside the definition of meaningfulness itself –, here they are seen as defining elements of meaningful work. However, Rosso et al. also suggest two further pathways, unification and individuation, which are not found in our definition and thus merit further inspection.

As regards unification, Rosso et al. (2010, p. 115) define it as actions that “bring individuals into harmony with other beings or principles.” Thus belongingness as interpersonal connectedness and closeness as well as social identification with others at work are at the heart of the unification pathway (pp. 111–112). However, in here we want to follow Pratt and Ashforth (2003) in making a distinction between meaningfulness *in* working and meaningfulness *at* work, with the former referring to the degree that the tasks and work conducted is meaningful, and the latter to one's “membership in the organization” and whom one surrounds oneself with as part of this membership. We see that while belongingness and unification are very closely aligned with the latter, meaningfulness at work, they probably would not contribute much to the degree that individuals view the work itself as meaningful^4^. We see that “meaningful work” as such refers mainly to meaningfulness in working, to the degree that what one does at work is meaningful. Thus we would not include unification and belongingness in our definition of meaningful work. However, this conclusion depends to a large degree on how we define “work” in meaningful work. If one includes the work community into one's definition of work, then belongingness indeed could be seen as at least an important source of significance and meaningfulness.

The fourth and final pathway suggested by Rosso et al. (2010, p. 115) is *individuation*, which they define as being about “actions that define and distinguish the self as valuable and worthy.” Defined as such, it thus seems to align closely with what is here called significance, which is seen as an overall evaluation of the intrinsic value of work, instead of a mere pathway to it. However, in addition to this significance dimension, their understanding of individuation also includes self-efficacy as the ability to produce an intended effect. Should we consider self-efficacy as a dimension of meaningful work? Even though there is some merit in this suggestion, we see that self-efficacy alone is not enough for significance. Even if one would be very effective in accomplishing certain things at work, one can see the work as completely meaningless and insignificant, if the accomplishments don't align at all with who one is and the goals the accomplishments serve are not connected to anything of real value. Borrowing an example that has been much used within philosophy, Sisyphus—the antihero from Greek mythology—pushing the same rock up the same hill (or pushing more and more similar rocks up that hill) can be very competent and effective in his activity, but still this activity is taken to be a paradigmatic case of meaningless action (Taylor, 1988) as it doesn't contribute to anything of value. Accordingly, we would argue that a sense of self-efficacy might be an important source of meaning that, when high, can strengthen one's sense of self-realization and broader purpose at work. But alone it is not enough to make an activity or work meaningful.

### How proposed sources of meaningful work connect to self-realization and broader purpose

One advantage of a separation between self-realization and broader purpose as two types of intrinsic value defining what makes work significant is that we can look at various proposed sources of meaningful work and make predictions about how they would relate to the two. We argue that several proposed sources of meaningful work connect to one of these elements stronger than the other. As our focus has been on the definition of meaningful work as such, we will not aim to offer any comprehensive account of various potential sources of meaningful work. Instead, we highlight a few factors as examples of how they are connected to either self-realization or broader purpose.

As regards self-realization, organizational practices and structures that give employees more freedom to decide their goals and how to pursue them, are arguably one important source. Accordingly, we suggest that autonomy from the job dimensions theory, defined as “the degree to which the job provides substantial freedom, independence, and discretion to the individual in scheduling the work and in determining the procedures to be used in carrying it out” (Hackman and Oldham, 1976, p. 258), should have a positive impact on employees' sense of self-realization. Similarly, *skill variety* which is about one being able to use “a number of different skills and talents” at work should have a positive impact on self-realization as it allows individual to bring a broader set of oneself and one's strengths into one's work performance. Also various forms of authentic behaviors (Ménard and Brunet, 2011) and person-job fit (Scroggins, 2008) have been associated with meaningful work, and we see that this association is due to the fact that the ability to engage in authentic behaviors and having a tighter person-job fit both increase one's sense of self-realization. Furthermore, Pratt and Ashforth (2003, p. 320) argue that the practices of employee involvement that empower individuals through sharing information, developing knowledge, rewarding skill acquisition and inviting participation most probably increase one's sense of meaningful work. What we suggest is that instead of improving meaningfulness as an undistinguished whole, they more specifically improve one's sense of self-realization.

What organizational factors would, in turn, influence employees' sense of higher purpose? Having a compelling mission—goals, values and purposes to which an organization is dedicated (Rosso et al., 2010)—is one strong candidate as such mission can help to communicate to the employee what is the higher purpose one is serving through one's work (Rosso et al., 2010). However, this can be a double-edged sword, because such strong mission can make the employees also more sensitive to actions that violate such mission. Task significance from job design theory, defined as “the degree to which the job has a substantial impact on the lives or work of other people” (Hackman and Oldham, 1976, p. 257) is also a natural candidate. The more the employees feel that their work has a positive impact in the lives of other people, the more they should feel that they are serving a higher purpose through their work. Accordingly, theory has suggested (Hackman and Oldham, 1976, 1980), and research has convincingly shown (see, Humphrey et al., 2007) that task significance and meaningful work are strongly associated. Here we make the more specific suggestion that having a compelling mission and task significance are especially connected to one's sense of broader purpose.

However, objective impact, to which task significance refers to, does not alone determine how much the employees experience that they are having a prosocial impact through their work. Also the salience of this impact plays a role as employees might be more or less aware of the impact they are making. Accordingly, Grant has shown that having a direct contact with the beneficiary increases people's sense of prosocial impact (e.g., Grant, 2008), and would most probably influence also their sense of serving a higher purpose through their work.

The list of factors discussed here is not meant to be exhaustive. There are probably many other factors that could influence employees' sense of self-realization and higher purpose. What our brief review has aimed to show is how various proposed sources of meaningful work are usually connected to either self-realization or broader purpose, and this explains why they are seen as contributing to meaningfulness in the first place. The natural next step is, of course, to start empirically to examine these and other connections.

## Discussion

Labels aside, there is an evaluation one can make about work—an evaluation where one looks at one's work and wonders whether it is intrinsically valuable and worth doing as such. We argue that this is a fundamental evaluation of work—or any other activity. The question of significance is not about the value of work from any particular perspective, but more generally whether it is worth engaging in. We call this general evaluation *significance*, and have argued that it consists of two sub-dimensions, self-realization and broader purpose. But in the end the question of whether we should define meaningful work as consisting exactly of these three dimensions is a secondary question. The primary point is that the three dimensions are important questions about work in their own right. Psychological research on “happiness” nowadays rarely engages in discussions of what are the “true' elements of happiness. Instead the researchers have seen it as more useful to study the proposed dimensions—positive affect, life satisfaction, psychological functioning—separately (e.g., Diener et al., 2010). Similarly, research on meaningful work would gain from studying the three identified dimensions of meaningful work separately.

In addition to making a clear separation between three constructs of meaningful work—significance, self-realization, and broader purpose—the main contribution of the review at hand is to offer an suggestion about their relations with each other. Lepisto and Pratt (2017) importantly separate between two perspectives on meaningful work: realization and justification, which correspond to what we call self-realization and significance (although with slightly different definitions). However, unlike Lepisto and Pratt, we don't see them as two separate perspectives, but rather suggest that they are key definitional dimensions of meaningful work. Furthermore, we argue for a different understanding of their relationship: We see significance as the broader evaluation of work, with self-realization being a key dimension that contributes to our sense of significance. Additionally, we propose that besides the two perspectives identified by Lepisto and Pratt, there is a third perspective on meaningful work that is equally important: broader purpose. Beyond general significance and self-realization, humans as compassionate beings (Barclay and van Vugt, 2015) thus arguably find meaningfulness from being able to contribute to other people and broader values, also through their work (Pessi, 2017).

We acknowledge that future research might arrive at different conclusions about the relations between the three dimensions of meaningful work. But even in that case the mere identification of the three dimensions contributes to research: What future research needs to do is to make a clear stand on which of the three elements they see as being elemental parts of meaningful work, and what they see their relationship to be. Only by being clear about what is included in the construct of meaningful work, and what is excluded, can we make progress in researching the potential sources and consequences of meaningfulness at work.

Furthermore, given our 3-fold distinction, what is most urgently needed is measuring scales that would make it possible to empirically examine these three elements separately to learn more about their interrelations and their mutual and separate antecedents. The scales currently in use include items that tap into all three of these dimensions without making clear separations between these aspects and thus making it impossible to know to which of these three elements certain proposed sources of meaningfulness are connected to. For example, the five items used by Bunderson and Thompson (2009) include both items related to significance (e.g., “The work that I do is important”) and to broader purpose (e.g., “The work that I do makes the world a better place”). It is worth noting that the Work as Meaning Inventory -scale constructed by Steger et al. (2012a) has one subscale called *Greater good motivations* that includes items such as “I know my work makes a positive difference in the world' and thus could tap relatively well into the broader purpose element of meaningfulness. Similarly, the Comprehensive Meaningful Work Scale developed by Lips-Wiersma and Wright (2012) have a *Serving Others* subscale tapping into serving a broader purpose, and *Expressing Full Potential* subscale coming close to what is here called self-realization. The *Meaningfulness* scale of May et al. (2004) includes items that tap into significance (e.g., “My job activities are significant to me”) and general meaningfulness (e.g., “My job activities are personally meaningful to me”) and thus blends together significance and meaningfulness items. Similarly, the *Job Diagnostic Survey* (Hackman and Oldham, 1974) includes a subscale for *experienced meaningfulness of work* that measures the degree to which the work is generally meaningful (e.g., “The work I do on this job is very meaningful to me”) and worthwhile and significant (“Most of the things I have to do on this job seem useless or trivial”). A second subscale, *task significance*, measures the degree to which the job has a substantial impact on the lives of other people (e.g., “This job is one where a lot of other people can be affected by how well the work gets done”), and thus taps into broader purpose, and a third subscale, *autonomy*, measuring the degree to which the job provides substantial freedom, independence, and discretion (e.g., “The job gives me considerable opportunity for independence and freedom in how I do the work”), comes relatively close to the present definition of self-realization, although it seems to concentrate more on the absence of external constraints than on the presence of a feeling of being able to realize oneself. These scales can thus offer a good starting point for exploring the elements of meaningful work, and the high correlation (0.78) between greater good motivation subscale and positive meaning subscale in Steger et al. (2012a) or the fact that experienced meaningfulness partially mediates the relations between task significance and various job outcomes (Humphrey et al., 2007) could offer some initial evidence about the relation between broader purpose and significance. However, as these scales have not been designed to adhere to the present definitions and thus have certain conceptual differences, it could be worthwhile to also develop a new scale that would more specifically target the presently defined three dimensions and the subscales of which would be tested from the beginning to be compatible with each other. Using scales that would have distinct items tapping self-realization, broader purpose, and general significance would make it possible to empirically separate the three suggested elements of meaningfulness and thus start researching their sources and interrelations. For example, it would be interesting to see whether two scales, one having only “meaningfulness” items and another having only “significance” items, would be so closely related as to be empirically indistinguishable. Furthermore, such scales would make it possible to examine whether we should see self-realization and broader purpose as two key antecedents to significance, or whether the three dimensions are so closely related as to make it more wise to treat them as three dimensions of the same overarching construct, meaningfulness.

An additional benefit of the clearer definition of meaningful work offered in this article is that it helps to distinguish meaningful work from its neighboring concepts, such as work-place spirituality (e.g., Saks, 2011), calling (e.g., Wrzesniewski et al., 1997), and self-transcendence (e.g., Koltko-Rivera, 2006) at work, which have received increasing attention not only as research topics but also in the more popular business literature. Popular discussions on ethics, new value creation, and deeper value at work and in business would all benefit from a clearer understanding of work meaningfulness. Also, discussions on everyday experiences of work—such as compassion fatigue and the risks of burnout in pursuing a calling (e.g., Bunderson and Thompson, 2009)—would gain from being connected to this understanding of meaningful work. We also believe that recognizing the three dimensions of meaningful work could be utilized in designing interventions that aim to promote meaningful work, and to ponder and explore which of these dimensions may be fostered from the outside (e.g., by supervisors at work) and what dimensions can only arise from individuals themselves. Such research would have far-reaching organizational applicability.

One key limitation has to be acknowledged as regards the current work. The articles we review and the discussions we engage with come almost exclusively from Western, mainly American, context. There is cross-cultural work showing that both self-realization and having a broader purpose are valued across cultural boundaries as important values (e.g., Chirkov et al., 2003; Schwartz et al., 2012; Feygina and Henry, 2015) and some work on the various meanings people attach to work across countries (e.g., Lundberg and Peterson, 1994), but much more research looking at work meaningfulness across cultures would be needed before any cross-cultural generalizations could be made. Thus an important future direction for research would be to examine whether the generalizations we and others have drawn about meaningful work would apply in other cultural contexts as well.

## Conclusion

Work has become a focal area “in providing meaning, stability, and a sense of community and identity” in people's lives (Cartwright and Holmes, 2006, p. 202). One could even argue that career has increasingly “taken the role of religion” in people's lives, thus compelling people to find more significance in their work than ever before (Baumeister and Vohs, 2002, p. 615). At the same time, recent rapid changes in working life due to technological developments represent a challenge for how to ensure meaningful work also in the future (Ford, 2015; Di Fabio and Blustein, 2016).

Accordingly, gaining a deeper understanding of what meaningful work is fundamentally about can assist us in building future workplaces that better address the existential needs of human beings. Here we have argued that when we talk about meaningful work, we talk about three separable components: The subjective experience of work as intrinsically *significant* and worth doing, the experience that one is able to *realize oneself* through work, and the work serving a *broader purpose*. The latter two are taken to be two key dimensions or types of intrinsic value that together define what makes work feel significant. In other words, if we are able to provide people with work where they can realize themselves and where they feel they are serving a broader purpose, we give people the opportunity to truly feel that their work is significant and worth doing.

## Author contributions

FM and AP conceptualized the article, and FM wrote the first version of the article. FM and AP together edited and finalized the article. FM and AP approved the final version and can both be held accountable for the content.

### Conflict of interest statement

The authors declare that the research was conducted in the absence of any commercial or financial relationships that could be construed as a potential conflict of interest.

## Footnotes

^1^ Along with others (Rosso et al., 2010; Lepisto and Pratt, 2017), we will use the terms meaningfulness of work and meaningful work interchangeably.

^2^ There were also a few idiosyncratic definitions that involved dimensions that occured only in one definition such as offering ”opportunities for eudaimonian activity” (Walsh, 1994, p. 244) or a sense of belonging (Schnell et al., 2013). However, here we concentrate on dimensions that received support from several different authors.

^3^ Here, as elsewhere in the article, our focus is on paid work and not, e.g., voluntary work.

^4^ It should be noted that research on meaning in life has shown how belongingness contributes to meaningfulness (see Lambert et al., 2013). This highlights an important difference between meaning in life and meaningful work. While life is not only about actions but also about just “being alive,” work is primarily seen as an action, something that people do. Thus being a part of a community through one's work can probably be important for one's sense of *meaning in life*, but not what people think about when they assess the *meaningfulness of their work*.

## References

[B1] Allan B. A. Autin K. L. Duffy R. D. (2014). Examining social class and work meaning within the psychology of working framework. J. Career Assess. 22, 543–561. 10.1177/1069072713514811

[B2] Allan B. A. Autin K. L. Duffy R. D. (2016). Self-determination and meaningful work: exploring socioeconomic constraints. Front. Psychol. 7:71. 10.3389/fpsyg.2016.00071 26869970 PMC4735399

[B3] Allan B. A. Duffy R. D. Collisson B. (2017). Helping others increases meaningful work: evidence from three experiments. J. Counsel. Psychol. 65, 155–165. 10.1037/cou0000228 28493738

[B4] Arneson R. J. (1987). Meaningful work and market socialism. Ethics 97, 517–545. 10.1086/292864

[B5] Arnold K. A. Turner N. Barling J. Kelloway E. K. McKee M. C. (2007). Transformational leadership and psychological well-being: the mediating role of meaningful work. J. Occup. Health Psychol. 12, 193–203. 10.1037/1076-8998.12.3.193 17638487

[B6] Arnoux-Nicolas C. Sovet L. Lhotellier L. Di Fabio A. Bernaud J. L. (2016). Perceived work conditions and turnover intentions: the mediating role of meaning of work. Front. Psychol. 7:704. 10.3389/fpsyg.2016.00704 27242616 PMC4863887

[B7] Bailey C. Madden A. Alfes K. Shantz A. Soane E. (2017). The mismanaged soul: existential labor and the erosion of meaningful work. Hum. Resour. Manage. Rev. 27, 416–430. 10.1016/j.hrmr.2016.11.001

[B8] Barclay P. van Vugt M. (2015). The evolutionary psychology of human prosociality: adaptations, byproducts, and mistakes, in The Oxford Handbook of Prosocial Behavior, eds Schroeder D. A. Graziano W. G. (Oxford: Oxford University Press), 37–60.

[B9] Baumeister R. F. (1991). Meanings of Life. New York, NY: The Guilford Press.

[B10] Baumeister R. F. Vohs K. D. (2002). The pursuit of meaningfulness in life, in Handbook of Positive Psychology, eds Snyder C. R. Lopez S. J. (New York, NY: Oxford University Press), 608–618.

[B11] Beadle R. Knight K. (2012). Virtue and meaningful work. Busin. Ethics Quart. 22, 433–450. 10.5840/beq201222219

[B12] Bellah R. N. Sullivan W. M. Tipton S. M. Swidler A. Madsen R. P. (1985). Habits of the Heart. Berkeley, CA: University of California Press.

[B13] Berg J. M. Dutton J. E. Wrzesniewski A. (2013). Job crafting and meaningful work, in Purpose and Meaning in the Workplace, eds Dik B. J. Byrne Z. S. Steger M. F. (Washington, DC: American Psychological Association), 81–104.

[B14] Berg J. M. Grant A. M. Johnson V. (2009). When callings are calling: crafting work and leisure in pursuit of unanswered occupational callings. Organ. Sci. 21, 973–994. 10.1287/orsc.1090.0497

[B15] Bowie N. E. (1998). A Kantian theory of meaningful work. J. Bus. Ethics 17, 1083–1092. 10.1023/A:1006023500585

[B16] Britt T. W. Adler A. B. Bartone P. T. (2001). Deriving benefits from stressful events: the role of engagement in meaningful work and hardiness. J. Occup. Health Psychol. 6, 53–63. 10.1037/1076-8998.6.1.53 11199257

[B17] Bunderson J. Thompson J. (2009). The call of the wild: zookeepers, callings, and the double-edged sword of deeply meaningful work. Adm. Sci. Q. 54, 32–57. 10.2189/asqu.2009.54.1.32

[B18] Camus A. (1955/2000). The absurdity of human existence, in The Meaning of Life, 2nd Edn, ed Klemke E. D. O'Brien J. (New York, NY: Oxford University Press), 94–100.

[B19] Cartwright S. Holmes N. (2006). The meaning of work: the challenge of regaining employee engagement and reducing cynicism. Hum. Resour. Manage. Rev. 16, 199–208. 10.1016/j.hrmr.2006.03.012

[B20] Chalofsky N. (2003). An emerging construct for meaningful work. Hum. Resour. Develop. Int. 6, 69–83. 10.1080/1367886022000016785

[B21] Chalofsky N. Cavallaro L. (2013). A good living versus a good life: meaning, purpose, and HRD. Adv. Devel. Hum. Resour. 15, 331–340. 10.1177/1523422313498560

[B22] Cheney G. Zorn T. E. Jr. Planalp S. Lair D. J. (2008). Meaningful work and personal/social well-being organizational communication engages the meanings of work. Ann. Int. Commun. Assoc. 32, 137–185. 10.1080/23808985.2008.11679077

[B23] Chirkov V. Ryan R. M. Kim Y. Kaplan U. (2003). Differentiating autonomy from individualism and independence: a self-determination theory perspective on internalization of cultural orientations and well-being. J. Pers. Soc. Psychol. 84, 97–110. 10.1037/0022-3514.84.1.97 12518973

[B24] Ciulla J. B. (2000). The Working Life: The Promise and Betrayal of Modern Work. New York, NY: Three Rivers Press.

[B25] Constantini A. Sartori R. Ceschi A. (2017). Framing workplace innovation through an organisational psychology perspective: a review of current WPI studies, in Workplace Innovation. Aligning Perspectives on Health, Safety and Well-Being, eds Oeij P. Rus D. Pot F. (Cham: Springer), 131–147.

[B26] Dempsey S. E. Sanders M. L. (2010). Meaningful work? Non-profit marketization and work/life imbalance in popular autobiographies of social entrepreneurship. Organization 17, 437–459. 10.1177/1350508410364198

[B27] Diener E. Ng W. Harter J. Arora R. (2010). Wealth and happiness across the world: material prosperity predicts life evaluation, whereas psychosocial prosperity predicts positive feeling. J. Person. Social Psychol. 99, 52–61. 10.1037/a0018066 20565185

[B28] Di Fabio A. Blustein D. L. (2016). Editorial: from meaning of working to meaningful lives: the challenges of expanding decent work. Front. Psychol. 7:1119. 10.3389/fpsyg.2016.01119 27512380 PMC4961706

[B29] Dik B. J. Duffy R. D. Eldridge B. M. (2009). Calling and vocation in career counseling: recommendations for promoting meaningful work. Profession. Psychol. 40, 625–632. 10.1037/a0015547

[B30] Fairlie P. (2011). Meaningful work, employee engagement, and other key employee outcomes: implications for human resource development. Adv. Devel. Hum. Resour. 13, 508–525. 10.1177/1523422311431679

[B31] Feygina I. Henry P. J. (2015). Culture and prosocial behavior, in The Oxford Handbook of Prosocial Behavior, eds Schroeder D. A. Graziano W. G. (Oxford: Oxford University Press), 188–208.

[B32] Ford M. (2015). Rise of the Robots: Technology and the Threat of a Jobless Future. New York, NY: Basic Books.

[B33] Frankl V. E. (2006/1946). Man's Search for Meaning. Boston, MA: Beacon Press.

[B34] Geldenhuys M. Łaba K. Venter C. M. (2014). Meaningful work, work engagement and organisational commitment. SA J. Ind. Psychol. 40, 1–10. 10.4102/sajip.v40i1.1098

[B35] George L. S. Park C. L. (2014). Existential mattering: bringing attention to a neglected but central aspect of meaning? in Meaning in Positive and Existential Psychology, eds Batthyany A. Russo-Netzer P. (New York, NY: Springer), 39–51.

[B36] George L. S. Park C. L. (2016). Meaning in life as comprehension, purpose, and mattering: toward integration and new research questions. Rev. General Psychol. 20, 205–220. 10.1037/gpr0000077

[B37] Giddens A. (1991). Modernity and Self-Identity. Stanford, CA: Stanford University Press.

[B38] Grant A. M. (2008). The significance of task significance: job performance effects, relational mechanisms, and boundary conditions. J. Appl. Psychol. 93, 108–124. 10.1037/0021-9010.93.1.108 18211139

[B39] Hackman J. R. Oldham G. R. (1974). The Job Diagnostic Survey: An Instrument for the Diagnosis of Jobs and the Evaluation of Job Redesign Projects. Technical Report No. 4, Department of Administrative Sciences, Yale University.

[B40] Hackman J. R. Oldham G. R. (1975). Development of the job diagnostic survey. J. Appl. Psychol. 60, 159–170. 10.1037/h0076546

[B41] Hackman J. R. Oldham G. R. (1976). Motivation through the design of work: test of a theory. Organ. Behav. Hum. Perform. 16, 250–279. 10.1016/0030-5073(76)90016-7

[B42] Hackman J. R. Oldham G. R. (1980). Work Redesign. Reading, MA: Addison Wesley.

[B43] Harlow L. L. Newcomb M. D. Bentler P. M. (1986). Depression, self-derogation, substance use, and suicide ideation: lack of purpose in life as a mediational factor. J. Clin. Psychol. 42, 5–21. 10.1002/1097-4679(198601)42:1<5::AID-JCLP2270420102>3.0.CO;2-9 3950015

[B44] Harris K. J. Kacmar K. M. Zivnuska S. (2007). An investigation of abusive supervision as a predictor of performance and the meaning of work as a moderator of the relationship. Leadersh. Q. 18, 252–263. 10.1016/j.leaqua.2007.03.007

[B45] Haybron D. M. (2008). The Pursuit of Unhappiness: The Elusive Psychology of Well-Being. New York, NY: Oxford University Press.

[B46] Heine S. J. Proulx T. Vohs K. D. (2006). The meaning maintenance model: on the coherence of social motivations. Person. Social Psychol. Rev. 10, 88–110. 10.1207/s15327957pspr1002_1 16768649

[B46a] Hirschi A. (2012). Callings and work engagement: Moderated mediation model of work meaningfulness, occupational identity, and occupational self-efficacy. J. Couns. Psychol. 59, 479–485. 10.1037/a0028949 22774870

[B47] Horowitz C. R. Suchman A. L. Branch W. T. Frankel R. M. (2003). What do doctors find meaningful about their work? Ann. Intern. Med. 138:772 10.7326/0003-4819-138-9-200305060-00028 12729445 PMC4303370

[B48] Humphrey S. E. Nahrgang J. D. Morgeson F. P. (2007). Integrating motivational, social, and contextual work design features: a meta-analytic summary and theoretical extension of the work design literature. J. Appl. Psychol. 92, 1332–1356. 10.1037/0021-9010.92.5.1332 17845089

[B49] Jelinek R. Ahearne M. (2010). Be careful what you look for: the effect of trait competitiveness and long hours on salesperson deviance and whether meaningfulness of work matters. J. Market. Theory Pract. 18, 303–321. 10.2753/MTP1069-6679180401

[B50] Kahn W. A. (1990). Psychological conditions of personal engagement and disengagement at work. Acad. Manage. J. 33, 692–724. 10.2307/256287

[B51] Kira M. Balkin D. B. (2014). Interactions between work and identities: thriving, withering, or redefining the self? Hum. Resour. Manage. Rev. 24, 131–143. 10.1016/j.hrmr.2013.10.001

[B52] Klinger E. (1998). The search for meaning in evolutionary perspective and its clinical implications, in The Human Quest For Meaning: A Handbook Of Psychological Research and Clinical Applications, eds Wong P. T. P. Fry P. S. (Mahwah, NJ: Lawrence Erlbaum Associates), 27–50.

[B53] Koltko-Rivera M. E. (2006). Rediscovering the later version of Maslow's hierarchy of needs: self-transcendence and opportunities for theory, research, and unification. Rev. General Psychol. 10, 302–317. 10.1037/1089-2680.10.4.302

[B54] Lambert N. M. Stillman T. F. Hicks J. A. Kamble S. Baumeister R. F. Fincham F. D. (2013). To belong is to matter: sense of belonging enhances meaning in life. Person. Soc. Psychol. Bull. 39, 1418–1427. 10.1177/0146167213499186 23950557

[B55] Lee G. K. Carter E. W. (2012). Preparing transition-age students with high-functioning autism spectrum disorders for meaningful work. Psychol. Sch. 49, 988–1000. 10.1002/pits.21651

[B56] Leiter M. P. Harvie P. Frizzell C. (1998). The correspondence of patient satisfaction and nurse burnout. Soc. Sci. Med. 47, 1611–1617. 10.1016/S0277-9536(98)00207-X 9823056

[B57] Lepisto D. A. Pratt M. G. (2017). Meaningful work as realization and justification toward a dual conceptualization. Organ. Psychol. Rev. 7, 99–121. 10.1177/2041386616630039

[B58] Leufstadius C. Eklund M. Erlandsson L. K. (2009). Meaningfulness in work - Experiences among employed individuals with persistent mental illness. Work J. Prevent. Assess. Rehabilit. 34, 21–32. 10.3233/WOR-2009-0899 19923673

[B59] Lieff S. J. (2009). Perspective: the missing link in academic career planning and development: pursuit of meaningful and aligned work. Acad. Med. 84, 1383–1388. 10.1097/ACM.0b013e3181b6bd54 19881426

[B60] Lips-Wiersma M. Morris L. (2009). Discriminating between “meaningful work”and the “management of meaning.” J. Business Ethics 88, 491–511. 10.1007/s10551-009-0118-9

[B61] Lips-Wiersma M. Wright S. (2012). Measuring the meaning of meaningful work development and validation of the comprehensive meaningful work scale (CMWS). Group Organ. Manage. 37, 655–685. 10.1177/1059601112461578

[B62] Lundberg C. D. Peterson M. F. (1994). The meaning of working in U.S. and Japanese local governments at three hierarchical levels. Hum. Relat. 47, 1459–1487. 10.1177/001872679404701202

[B63] Martela F. (2015). Fallible inquiry with ethical ends-in-view: a pragmatist philosophy of science for organizational research. Organ. Stud. 36, 537–563. 10.1177/0170840614559257

[B64] Martela F. (2017a). Can good life be measured? The dimensions and measurability of a life worth living, in Metrics of Subjective Well-Being: Limits and Improvements, eds Brulé G. Maggino F. (Cham: Springer), 21–42.

[B65] Martela F. (2017b). Meaningfulness as contribution. South. J. Philos. 55, 232–256. 10.1111/sjp.12217

[B66] Martela F. Ryan R. M. (2016a). The benefits of benevolence: basic psychological needs, beneficence, and the enhancement of well-being. J. Pers. 84, 750–764. 10.1111/jopy.12215 26249135

[B67] Martela F. Ryan R. M. (2016b). Prosocial behavior increases well-being and vitality even without contact with the beneficiary: causal and behavioral evidence. Motiv. Emot. 40, 351–357. 10.1007/s11031-016-9552-z

[B68] Martela F. Ryan R. M. Steger M. F. (2017). Meaning in life is more than happiness: autonomy, competence, relatedness and benevolence as consistent predictors of meaning. J. Happiness Stud. [Epub ahead of print]. 10.1007/s10902-017-9869-7.

[B69] Martela F. Steger M. F. (2016). The meaning of meaning in life: coherence, purpose and significance as the three facets of meaning. J. Positive Psychol. 11, 531–545. 10.1080/17439760.2015.1137623

[B70] May D. R. Gilson R. L. Harter L. M. (2004). The psychological conditions of meaningfulness, safety and availability and the engagement of the human spirit at work. J. Occupat. Organ. Psychol. 77, 11–37. 10.1348/096317904322915892

[B71] McKnight P. E. Kashdan T. B. (2009). Purpose in life as a system that creates and sustains health and well-being: an integrative, testable theory. Rev. General Psychol. 13, 242–251. 10.1037/a0017152

[B72] Ménard J. Brunet L. (2011). Authenticity and well-being in the workplace: a mediation model. J. Manag. Psychol. 26, 331–346. 10.1108/02683941111124854

[B73] Menges J. I. Tussing D. V. Wihler A. Grant A. M. (2017). When job performance is all relative: how family motivation energizes effort and compensates for intrinsic motivation. Acad. Manage. J. 60, 695–719. 10.5465/amj.2014.0898

[B74] Michaelson C. (2005). Meaningful motivation for work motivation theory. Acad. Manage. Rev. 30, 235–238. 10.5465/AMR.2005.16387881

[B75] Michaelson C. (2011). Whose responsibility is meaningful work? J. Manage. Devel. 30, 548–557. 10.1108/02621711111135152

[B76] Michaelson C. Pratt M. G. Grant A. M. Dunn C. P. (2014). Meaningful work: connecting business ethics and organization studies. J. Business Ethics 121, 77–90. 10.1007/s10551-013-1675-5

[B77] Molden D. C. Dweck C. S. (2006). Finding“ meaning” in psychology: a lay theories approach to self-regulation, social perception, and social development. Am. Psychol. 61, 192–203. 10.1037/0003-066X.61.3.192 16594836

[B78] Munn S. L. (2013). Unveiling the work–life system: the influence of work–life balance on meaningful work. Adv. Devel. Hum. Resour. 15, 401–417. 10.1177/1523422313498567

[B79] Park C. L. (2010). Making sense of the meaning literature: an integrative review of meaning making and its effects on adjustment to stressful life events. Psychol. Bull. 136, 257–301. 10.1037/a0018301 20192563

[B80] Pavlish C. Hunt R. (2012). An exploratory study about meaningful work in acute care nursing. Nursing Forum 47, 113–122. 10.1111/j.1744-6198.2012.00261.x 22512769

[B81] Pessi A. B. (2013). Privatized religiosity revisited: building an authenticity model of individual–church relations. Soc. Comp. 60, 3–21. 10.1177/0037768612472592

[B82] Pessi A. B. (2017). Dazed and amazed by moonlight – exploring sense of meaning as the mediator of the effects of religion, belonging, and benevolence on wellbeing. Nordic J. Religion Society 30, 24–42. 10.18261/issn.1890-7008-2017-01-02

[B83] Pratt M. G. (2000). The good, the bad, and the ambivalent: managing identification among Amway distributors. Adm. Sci. Q. 45, 456–493. 10.2307/2667106

[B84] Pratt M. G. Ashforth B. E. (2003). Fostering meaningfulness in working and at work, in Positive Organizational Scholarship: Foundations of a New Discipline, eds Cameron K. S. Dutton J. E. Quinn R. E. (San Francisco, CA: Berrett-Koehler), 309–327.

[B85] Raub S. Blunschi S. (2014). The power of meaningful work: how awareness of CSR initiatives fosters task significance and positive work outcomes in service employees. Cornell Hospit. Quart. 55, 10–18. 10.1177/1938965513498300

[B86] Renn R. W. Vandenberg R. J. (1995). The critical psychological states: an underrepresented component in job characteristics model research. J. Manage. 21, 279–303. 10.1177/014920639502100206

[B87] Roessler B. (2012). Meaningful work: arguments from autonomy. J. Political Philos. 20, 71–93. 10.1111/j.1467-9760.2011.00408.x

[B88] Rosso B. D. Dekas K. H. Wrzesniewski A. (2010). On the meaning of work: a theoretical integration and review. Res. Organ. Behav. 30, 91–127. 10.1016/j.riob.2010.09.001

[B89] Rothmann S. Hamukang'andu L. (2013). Callings, work role fit, psychological meaningfulness and work engagement among teachers in Zambia. South Afr. J. Educ. 33, 1–16. 10.15700/saje.v33n2a699

[B90] Saks A. M (2011). Workplace spirituality and employee engagement. J. Manage. Spirit. Religion 8, 317–340. 10.1080/14766086.2011.630170

[B91] Sarros J. C. Tanewski G. A. Winter R. P. Santora J. C. (2002). Work alienation and organizational leadership. Br. J. Manage. 13, 285–304. 10.1111/1467-8551.00247

[B92] Sayer A. (2009). Contributive justice and meaningful work. Res Publica 15, 1–16. 10.1007/s11158-008-9077-8

[B93] Schnell T. Höge T. Pollet E. (2013). Predicting meaning in work: theory, data, implications. J. Posit. Psychol. 8, 543–554. 10.1080/17439760.2013.830763

[B94] Schnell T. Hoof M. (2012). Meaningful commitment: finding meaning in volunteer work. J. Beliefs Values 33, 35–53. 10.1080/13617672.2012.650029

[B95] Schwartz A. (1982). Meaningful work. Ethics 92, 634–646. 10.1086/292380

[B96] Schwartz S. H. Cieciuch J. Vecchione M. Davidov E. Fischer R. Beierlein C.. (2012). Refining the theory of basic individual values. J. Pers. Soc. Psychol. 103, 663–688. 10.1037/a0029393 22823292

[B97] Scroggins W. A. (2008). Antecedents and outcomes of experienced meaningful work: a person-job fit perspective. J. Business Inquiry 7, 68–78.

[B98] Silberman I. (2005). Religion as a meaning system: implications for the new millennium. J. Soc. Issues. 61, 641–663. 10.1111/j.1540-4560.2005.00425.x

[B99] Sparks J. R. Schenk J. A. (2001). Explaining the effects of transformational leadership: an investigation of the effects of higher-order motives in multilevel marketing organizations. J. Organ. Behav. 22, 849–869. 10.1002/job.116

[B100] Spreitzer G. M. (1996). Social structural characteristics of psychological empowerment. Acad. Manage. J. 39, 483–504. 10.2307/256789

[B101] Starr S. (2008). Authenticity: a concept analysis. Nursing Forum 43, 55–62. 10.1111/j.1744-6198.2008.00096.x 18447890

[B102] Steers R. M. Mowday R. T. Shapiro D. L. (2005). Meaningful motivation for work motivation theory - response. Acad. Manage. Rev. 30, 238–238. 10.5465/AMR.2005.16387882

[B103] Steger M. F. (2012). Experiencing meaning in life - Optimal functioning at the nexus of well-being, psychopathology, and spirituality, in The Human Quest for Meaning: Theories, Research, and Applications, 2nd Edn, ed Wong P. T. P. (New York, NY: Routledge), 165–184.

[B104] Steger M. F. Dik B. J. (2009). If one is looking for meaning in life, does it help to find meaning in work? Appl. Psychol. Health Well Being 1, 303–320. 10.1111/j.1758-0854.2009.01018.x

[B105] Steger M. F. Dik B. J. Duffy R. D. (2012a). Measuring meaningful work the work and meaning inventory (WAMI). J. Career Assess. 20, 322–337. 10.1177/1069072711436160

[B106] Steger M. F. Frazier P. Oishi S. Kaler M. (2006). The meaning in life questionnaire: assessing the presence of and search for meaning in life. J. Couns. Psychol. 53, 80–93. 10.1037/0022-0167.53.1.80

[B107] Steger M. F. Littman-Ovadia H. Miller M. Menger L. Rothmann S. (2012b). Engaging in work even when it is meaningless: positive affective disposition and meaningful work interact in relation to work engagement. J. Career Assess. 21, 348–361. 10.1177/1069072712471517

[B108] Strong S. (1998). Meaningful work in supportive environments: experiences with the recovery process. Am. J. Occupat. Ther. 52, 31–38. 10.5014/ajot.52.1.31 9426856

[B109] Tanno K. Sakata K. Ohsawa M. Onoda T. Itai K. Yaegashi Y.. (2009). Associations of ikigai as a positive psychological factor with all-cause mortality and cause-specific mortality among middle-aged and elderly Japanese people: findings from the Japan collaborative cohort study. J. Psychosom. Res. 67, 67–75. 10.1016/j.jpsychores.2008.10.018 19539820

[B110] Taylor C. (1991). The Ethics of Authenticity. Cambridge, MA: Harvard University Press.

[B111] Taylor R. (1988). The meaning of life, in Life and Meaning: A Philosophical Reader, ed Hanfling O. (Oxford: Blackwell), 39–48.

[B112] Tech Times (2015, August 27). Machine simulates minimum wage earnings by turning a hand-crank for pennies. Tech Times. Availabel online at: http://www.techtimes.com/articles/79915/20150827/machine-simulates-minimum-wage-earnings-turning-hand-crank-pennies.htm.

[B113] Thomas K. W. Velthouse B. (1990). Cognitive elements of empowerment: an “interpretive” model of intrinsic task motivation. Acad. Manage. Rev. 15, 666–681.

[B114] Tolstoy L. (2000). My confession, in The Meaning of Life, 2nd Edn, ed Klemke E. D. Wierner L. (New York, NY: Oxford University Press), 11–20.

[B115] Tongeren D. R. V. Green J. D. Davis D. E. Hook J. N. Hulsey T. L. (2016). Prosociality enhances meaning in life. J. Posit. Psychol. 11, 225–236. 10.1080/17439760.2015.1048814

[B116] Tummers L. G. Knies E. (2013). Leadership and meaningful work in the public sector. Public Adm. Rev. 73, 859–868. 10.1111/puar.12138

[B117] Walsh A. J. (1994). Meaningful work as a distributive good. South. J. Philos. 32, 233–250. 10.1111/j.2041-6962.1994.tb00713.x

[B118] Weber M. (1958). The Protestant Ethic and the Spirit of Capitalism, ed Parsons T. New York, NY: Charles Scribner's Sons.

[B119] Wong P. T. (1998). Implicit theories of meaningful life and the development of the personal meaning profile (PMP), in The Human Quest for Meaning: A Handbook of Psychological Research and Clinical Applications, eds Wong P. T. P. Fry P. S. (Mahwah, NJ: Lawrence Erlbaum), 111–140.

[B120] Wrzesniewski A. McCauley C. Rozin P. Schwartz B. (1997). Jobs, careers, and callings: people's relations to their work. J. Res. Pers. 31, 21–33. 10.1006/jrpe.1997.2162

[B121] Yeoman R. (2014). Conceptualising meaningful work as a fundamental human need. J. Business Ethics 125, 235–251. 10.1007/s10551-013-1894-9

